# The relationship between perceived income inequality, adverse mental health and interpersonal difficulties in UK adolescents

**DOI:** 10.1111/jcpp.13719

**Published:** 2022-11-14

**Authors:** Blanca Piera Pi‐Sunyer, Jack L. Andrews, Amy Orben, Lydia G. Speyer, Sarah‐Jayne Blakemore

**Affiliations:** ^1^ Department of Psychology University of Cambridge Cambridge UK; ^2^ Institute of Cognitive Neuroscience University College London London UK; ^3^ School of Psychology UNSW Sydney New South Wales Australia; ^4^ MRC Cognition and Brain Sciences Unit University of Cambridge Cambridge UK

**Keywords:** Adolescence, mental health, interpersonal difficulties, development, poverty, socioeconomic status, perceived wealth inequalities

## Abstract

**Background:**

Adolescence is a period of life when young people increasingly define themselves through peer comparison and are vulnerable to developing mental health problems. In the current study, we investigated whether the subjective experience of economic disadvantage among friends is associated with social difficulties and poorer mental health in early adolescence.

**Methods:**

We used latent change score modelling (LCSM) on data from the UK Millennium Cohort Study, collected at ages 11 and 14 (*N* = 12,995). Each LCSM modelled the mean of an outcome related to mental health and interpersonal difficulties at age 11 (including self‐esteem, well‐being, emotional difficulties, peer problems, bullying, victimisation and externalising difficulties), the change of the outcome from ages 11 to 14 and its predictors, including perceived income inequality among friends (i.e. perceiving oneself as belonging to a poorer family than the families of one's friends).

**Results:**

Perceived income inequality predicted adverse mental health and a range of interpersonal difficulties during adolescence, even when controlling for objective family income. Follow‐up analyses highlighted that, at 11 years, young people who perceived themselves as belonging to poorer families than their friends reported worse well‐being, self‐esteem, internalising problems, externalising problems and victimisation at the same age (relative to those who perceived themselves as richer than or equal to their friends, or who did not know). Longitudinal analyses suggested that victimisation decreased from ages 11 to 14 to a greater extent for adolescents who perceived themselves as poorer than other adolescents.

**Conclusions:**

The salience of economic inequalities in proximal social environments (e.g. among friends) in early adolescence could further amplify the negative effects of economic disadvantage on mental health and behavioural difficulties during this period.

## Introduction

The socioeconomic (SES) gradient in health is a well‐established finding: individuals from low SES backgrounds, a combined measure of an individual's economic and social position, are more likely to experience mental and physical health problems, as well as poorer educational and cognitive outcomes, across the lifespan (Braveman, Egerter, & Williams, 2011; Dalmaijer et al., 2021; Lynch, Kaplan, & Shema, 1997). Income inequality, which refers to the ‘economic distance’ between the richest and poorest within a defined group of people, is also known to play an important role in perpetuating poorer outcomes. Children, adolescents and adults from countries with greater levels of income inequality, across SES groups, have poorer outcomes on indices including life expectancy, infant mortality, mental health and well‐being and peer victimisation, compared with individuals from more equal societies (Elgar et al., 2015, 2019; Pickett & Wilkinson, 2010, 2007). It has also been suggested that this extends to more proximal environments with high economic distance. For example, at the neighbourhood level, young boys from disadvantaged backgrounds who live among more affluent peers show more conduct problems and substance use than boys living in low‐income contexts (Odgers, Donley, Caspi, Bates, & Moffitt, 2015; Rivenbark et al., 2020).

While such studies have primarily used objective measures to index levels of SES and inequality, there is also evidence that the subjective experience of one's relative position in society impacts well‐being. For example, studies capturing individuals' perception of their relative social position in society, or subjective social status (SSS), have shown that SSS is a significant predictor of mental health symptoms (Quon & McGrath, 2014; Scott et al., 2014) and externalising behaviour (see Highlander & Jones, 2021 for review), even when controlling for income. In one study using data collected for a US national survey (NCS‐A) of nearly 6,500 adolescents aged 13–17 years, SSS predicted several adverse outcomes: individuals who perceived themselves as lower in status reported worse mental health‐related outcomes, including more mood and anxiety disorders, as well as substance use and behavioural disorders, relative to those who perceived themselves as higher status (McLaughlin, Costello, Leblanc, Sampson, & Kessler, 2012). This was true even when controlling for measures of SES such as parental income.

The effect of perceiving oneself as less well‐off than others may have disproportionate effects on health and well‐being at different points in life. For example, during adolescence, the period of life between 10 and 24 years (Sawyer, Azzopardi, Wickremarathne, & Patton, 2018), social evaluation becomes especially salient (Somerville, 2013). At the same time, individuals become better at taking the perspective of others and typically judge themselves less favourably when they compare themselves to others (Van der Aar, Peters, & Crone, 2018). Coupled with this, adolescents are exposed to shifting social environments (e.g. the move from primary to secondary school) and are at a heightened risk for developing mental health problems, with 75% of all mental health problems first emerging before the age of 24 (Andrews, Ahmed, & Blakemore, 2021; Kessler et al., 2007).

Given the important role that social comparisons play during adolescence, we sought to understand the extent to which proximal perceived income inequality (e.g. perceiving oneself as belonging to a poorer family than the families of one's friends) affects outcomes during this developmental stage. This is distinct from previous work, the majority of which has focused on more distal comparisons, for example, comparing oneself with others within a country or larger community (e.g. Scott et al., 2014). We used data from the UK Millennium Cohort Study (MCS), collected at ages 11 and 14, to investigate whether young adolescents' perceived income inequality (relative to friends) explains differences in trajectories of mental health and interpersonal difficulties, above any differences explained by objective measures of family income (Hypothesis 1a). If this hypothesis was supported, we further investigated whether adolescents who perceived their family as poorer than their friends' families at age 11 experience less favourable outcomes at age 11 (Hypothesis 1b) and show increasing difficulties across all outcomes between ages 11 and 14 (Hypothesis 1c), relative to participants who did not perceive themselves as poorer than their friends (see study preregistration in the Open Science Framework; https://osf.io/vfksd).

## Method

### Participants

Our study used data from the MCS, a population‐representative cohort study of 19,517 children born between the years 2000 and 2002 in the United Kingdom (Connelly & Platt, 2014). Seven waves of data have been collected to date, from when cohort members were aged 9 months to 17 years. In the current study, we analysed data from when cohort members were aged 11 (*n* = 13,469) and 14 (*n* = 11,872).

The sample of this study comprised 12,995 (female = 6491; male = 6505) adolescents who provided data about their perceived income inequality at age 11 (see Predictor Measures below). One participant was excluded due to missing data for weighting corrections for stratified clustered sampling. Eighty‐three per cent of the children in the sample identify as White, 7% as Pakistani/Bangladeshi, 3.2% as Black, 3% as Mixed, 2.5% as Indian and 1.3% as Other.

### Ethical considerations

Data were obtained from the UK Data Archive, University of Essex, in July 2020. The MCS has been approved by the UK National Health Service Research Ethics (see Shepherd & Gilbert, 2019). Written informed consent was obtained from all parents, as well as assent from cohort members, at both sweeps.

### Predictor measures

*Perceived Income Inequality Among Friends (PIIAF)* was measured by asking participants at age 11 to rate whether they perceived themselves as richer, poorer or the same as their friends, or whether they did not know. Most participants (*n =* 9,302; 71.6%) perceived themselves as the same as their friends, while 4.1% (*n* = 527) and 8.0% (*n* = 1,044) perceived themselves as poorer or richer, respectively, and 16.3% (*n* = 2,100) did not know. A power simulation on 500 unbalanced samples suggested these group sizes were sufficient to detect an effect at >99% power at alpha = .05 (see more details in Appendix S1).

*Objective family income* was measured using the participants' equalised weekly family income (EFI) at age 11, a measure of net disposable income which accounts for the differences in household size and composition across the United Kingdom. This measure ranged from £65.85 to £1,136.45, with the interquartile range of EFI falling between £258.27 and £536.61 (25th and 75th centiles).

### Outcome measures

*Well‐being* was measured with six items reflecting children's feelings about different parts of their life, including appearance, school, family and friends (see Appendix S2). Responses for each item (on a 7‐point scale) were aggregated such that a higher aggregate score denoted better overall well‐being (min = 6, max = 42). The items showed good internal consistency at ages 11 (Cronbach's alpha = .83) and 14 (Cronbach's alpha = .86).

*Self‐esteem* was measured with an abbreviated version of the self‐report 10‐item Rosenberg's Self‐Esteem Scale (Robins, Hendin, & Trzesniewski, 2001; see Appendix S3). Participants were presented with five statements reflecting positive feelings about the self, and responses for each item (on a 5‐point scale) were aggregated such that a higher score indicated higher self‐esteem (min = 5, max = 20). The items showed good internal consistency at ages 11 (Cronbach's alpha = .74) and 14 (Cronbach's alpha = 0.91).

*Internalising* and *externalising difficulties* were measured using the parent‐reported Strengths and Difficulties Questionnaire (SDQ; Goodman & Goodman, 2009; see Appendix S4). The SDQ includes five 5‐item subscales. The emotional difficulties and peer problems subscales were combined to produce an internalising behaviour score, and the conduct problems and hyperactivity subscales were combined to produce an externalising behaviour score. The prosocial behaviour scale was excluded. Responses for each item (on a 3‐point scale) were aggregated such that higher aggregate scores indicated more internalising or externalising difficulties (min = 0, max = 20). All SDQ subscales show longitudinal and gender invariance in the MCS (Murray, Speyer, Hall, Valdebenito, & Hughes, 2022), and the internalising and externalising scores have been previously validated (Kersten et al., 2016). Preregistered supplemental analyses were also conducted on the emotional difficulties and peer problems subscales separately (see supplemental analyses Appendices SA and SB).

*Bullying* (perpetration) and *victimisation* (being victimised) were measured using self‐report single‐item questions asking participants to report how often they pick on other children, or have been picked on by other children, both on a 6‐point scale (1 = most days, 6 = never; see Appendix S5). At age 14, another single‐item question was included for bullying and victimisation *online*. The two scales (physical or online) were aggregated and then averaged so that scores would be on the same scale as for age 11. The scores were reverse coded for both sweeps, such that higher scores corresponded to bullying more often or being victimised more often, respectively (min = 0, max = 5).

*Drinking* was measured using a self‐report single‐item question asking participants to report how many alcoholic drinks they had in the past month using a 7‐point scale (1 = never and 7 = 40 or more times). *Binge drinking* was measured using a self‐report single‐item question asking participants to report how many times they have had 5 or more alcoholic drinks in one sitting on a 6‐point scale (1 = never and 6 = 10 or more times; see Appendix S6). Higher scores corresponded to more drinking and binge drinking. Most participants reported no drinking or binge drinking behaviour both at ages 11 (97% and 99%, respectively) and 14 (78% and 91%, respectively). Analyses were therefore included in the Supporting Information and should be interpreted with caution due to low numbers (see supplemental analyses Appendices SC and SD).

### Analysis strategy

**Figure 1:**
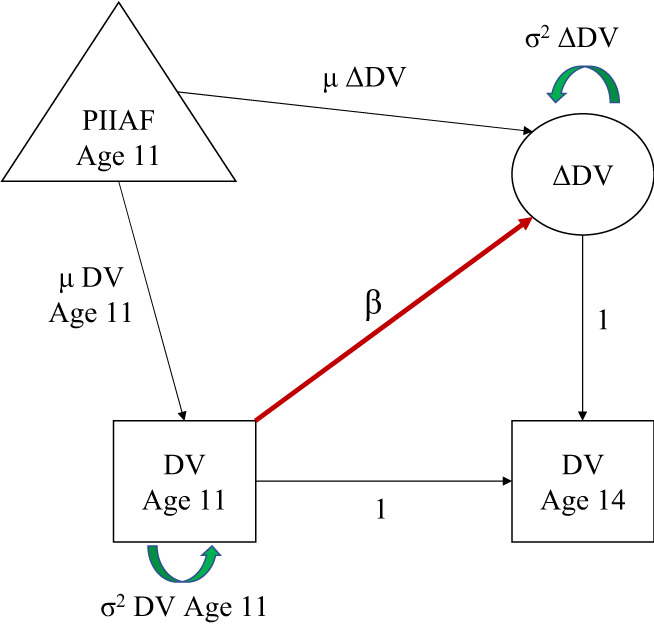
Latent change score model. *Note*. Variable DV is measured at ages 11 and 14 (DV Age 11 and DV Age 14). Change (ΔDV) between ages 11 and 14 is modelled as the latent variable. The LCSM estimates the effect of perceived income inequality (PIIAF) grouping on (A) the mean DV Age 11 (μ DV Age 11), (B) the variance of DV Age 11 (σ^2^ DV Age 11), (C) the mean of the change from ages 11 to 14 (μ ΔDV), (D) the variance of the change in DV (σ^2^ΔDV) and (E) The regression parameter β estimating ΔDV from DV Age 11. Control variables were included as exogenous predictors of the regressions on μ DV Age 11 and μ ΔDV. Adapted from Kievit et al. (2018) [Color figure can be viewed at wileyonlinelibrary.com]

We fitted a set of preregistered Latent Change Score Models (LCSM; Kievit et al., 2018; McArdle, 2009) for each of our outcome measures (10 in total; see preregistered project in https://osf.io/sv72e/). A LCSM is a class of Structural Equation Model that allows us to model a variety of parameters that detail within‐subject changes over time (see Figure 1). Any participant (*i*) has two outcome measurements at age 11 (DV_*i,*11_) and 14 (DV_*i,*14_), and a change score of DV_*i,*14_ *–*DV_*i,*11_ (*Δ*DV_*i*_). The model allows us to estimate the mean and variance at age 11 (*μ*DV_*i,*11_ and *σ* ^*2*^DV_*i,*11_), the mean and variance of the change score from ages 11 to 14 (*μΔ*DV_*i*_ and *σ* ^*2*^ *Δ*DV_*i*_) and the self‐feedback parameter (β; how much the change score *μΔ*DV_*i*_ is dependent on the scores at age 11 *μ*DV_*i,*11_).

In addition to these parameters, we included time‐invariant exogenous control variables in our model: objective family income, gender and ethnicity, all measured at age 11. We modelled objective family income on the log scale to better approximate a normal distribution, and dummy coded gender (2 levels) and ethnicity (6 levels). We constrained the control variables' regression onto both *μ*DV_*i,*11_ and *μΔ*DV_*i*_ to be equal across different groups of PIIAF. We further constrained the covariances of the control variables to be equal across PIIAF groups.

We estimated the models using the *lavaan* software package in R (Rosseel, 2012), and used full information maximum likelihood and robust maximum likelihood estimation to account for missingness and nonnormality. We treated all outcome measures as continuous variables. We compared models using Satorra–Bentler‐corrected *χ* ^2^ difference testing (alpha = .05; see Satorra & Bentler, 2010) and sample size adjusted Bayesian information criterion (as for large samples, even trivial differences may become significant in *χ* ^2^ difference testing; ΔBIC > 10; Schermelleh‐Engel, Moosbrugger, & Muller, 2003). Further, we used root mean square error of approximation (RMSEA < .05), comparative fit index (CFI > .90), Tucker–Lewis index (TLI > .90) and standardised root mean square residual (SRMR < .10) to assess the model fit (Kline, 2015). All models were additionally probed for robustness using the MCS survey weights and a more proximal (country‐level) measure of objective family income (see Appendices S7 and S8 as well as Figure S1).

#### Hypothesis 1a

To test our first hypothesis, we evaluated two multigroup LCSMs for each of our 10 outcome measures, with PIIAF as a grouping variable (groups: richer, poorer, same and I do not know). The first model allowed the five parameters (mean at age 11 *μ*DV_11_, variance at age 11 *σ* ^*2*^DV_11_, mean of the change score *μΔ*DV, variance of the change score *σ* ^*2*^ *Δ*DV and the self‐feedback parameter β) to vary between adolescents of different PIIAF groups. The second model constrained the same five parameters to be equal across PIIAF groups. If the first model fit the data substantially better, this was interpreted as PIIAF improving the model, therefore supporting our hypothesis. If hypothesis 1a was supported, we tested a series of exploratory model comparisons assessing whether freeing each of the five parameters to vary by PIIAF would improve model fit (see details in Appendix S9). In addition, if hypothesis 1a was supported, we tested two further hypotheses, as follows.

#### Hypotheses 1b (H1b) and 1c (H1c)

We ran a set of ANOVAs to test whether those who perceived themselves as belonging to poorer families than their friends (poorer PIIAF) fared worse on the outcome measures at age 11 (H1b), and on the change in the measures from ages 11 to 14 (H1c), than those in different PIIAF groups. These tests were performed where the best‐fitting model for an outcome measure allowed the mean score at age 11 *μ*DV_11_, or the mean of the change score *μΔ*DV, to vary freely with PIIAF respectively. In these cases, ANOVAs were run on the mean scores at age 11, or on the difference scores from ages 11 to 14 (mean age 14–mean age 11). In addition, to assess differences between the PIIAF groups (i.e. richer, equal, poorer and I do not know), we ran Bonferroni‐corrected post hoc pairwise comparisons. Hypothesis 1b was supported if adolescents in the poorer PIIAF group reported more difficulties at age 11 than adolescents in the other PIIAF groups. Hypothesis 1c was supported if adolescents in the poorer PIIAF group reported a greater increase in difficulties between ages 11 and 14 than adolescents in the other PIIAF groups.

We initially preregistered to use the InformativeTesting function in *lavaan* to examine this, however, the function could not handle a model of this complexity and did not converge properly across all outcome measures, hence, we decided to use ANOVAs instead.

## Results

### Hypothesis 1a

A series of *χ* ^2^ difference tests and BIC model comparisons suggested that LCSMs freeing all parameters to vary with PIIAF explained all our outcome variables better than the control models without this grouping variable, lending support to the hypothesis that PIIAF explains differences in well‐being, self‐esteem, internalising difficulties, externalising difficulties, bullying and victimisation trajectories (H1a; see Table 1 for *χ* ^2^ and BIC results for each outcome measure; see Tables S1 and S2 for control models). This suggests that whether an individual perceived themselves as poorer, richer or equal to their friends at age 11, or they did not know, explained the trajectories of their mental health and interpersonal difficulties over and above objective family income. See the Supporting Information for preregistered supplemental analyses of emotional difficulties and peer problems, as well as analyses of drinking and binge drinking. The latter were moved to the Supporting Information because both variables had little to no variance and results should be interpreted with caution.

**Table 1:** Results for χ^2^ difference tests and BIC model comparisons for each outcome variable

Outcome measure	Δχ^2^	*Δdf*	*p*‐Value	ΔBIC
Well‐being	194.04	15	<.001	191
Self‐esteem	259.63	15	<.001	222
Internalising difficulties	184.64	15	<.001	171
Externalising difficulties	113.68	15	<.001	48
Bullying	159.98	15	<.001	309
Victimisation	313.07	15	<.001	283

*χ* ^2^ and BIC results show that an LSCM freeing all parameters to vary with PIIAF fits each outcome measure better than an LSCM constraining all parameters to be the same across PIIAF.

All models fit the data well (all RMSEAs < .038; all CFIs > .985; all SRMRs < .036 and all TLI > .980; see Table S3 for model fit statistics). Additionally, we ran a series of preregistered exploratory model comparisons freeing different model parameters to vary with PIIAF to determine the best‐fitting model for each outcome variable (see details of best‐fitting models in Appendix S10).

### Hypothesis 1b

We ran a set of one‐way ANOVAs to test the hypothesis that individuals who perceive themselves as poorer than their friends would report more difficulties relative to all other PIIAF groups (i.e. richer, equal and I do not know) at age 11 (H1b). The ANOVAs showed a small but significant impact of PIIAF on well‐being, self‐esteem, internalising difficulties, externalising difficulties, bullying and victimisation, at age 11 (see Table 2 for ANOVA results for each outcome measure; see all mean scores of the outcomes measures in Table S4).

**Table 2:** ANOVA results for each outcome measure at age 11

Outcome measure	*F*	*df*s	*p*‐Value	η_p_^2^
Well‐being	66.97	3,12677	<.001	.02
Self‐esteem	62.20	3,12148	<.001	.02
Internalising difficulties	53.87	3,12418	<.001	.01
Externalising difficulties	45.40	3,12394	<.001	.01
Bullying	20.33	3,12864	<.001	.01
Victimisation	71.83	3,12860	<.001	.02

*F* tests and the corresponding effect size ($\eta_{p}^{2}$) resulting from the one‐way ANOVAs conducted for each outcome measure at age 11.

**Figure 2:**
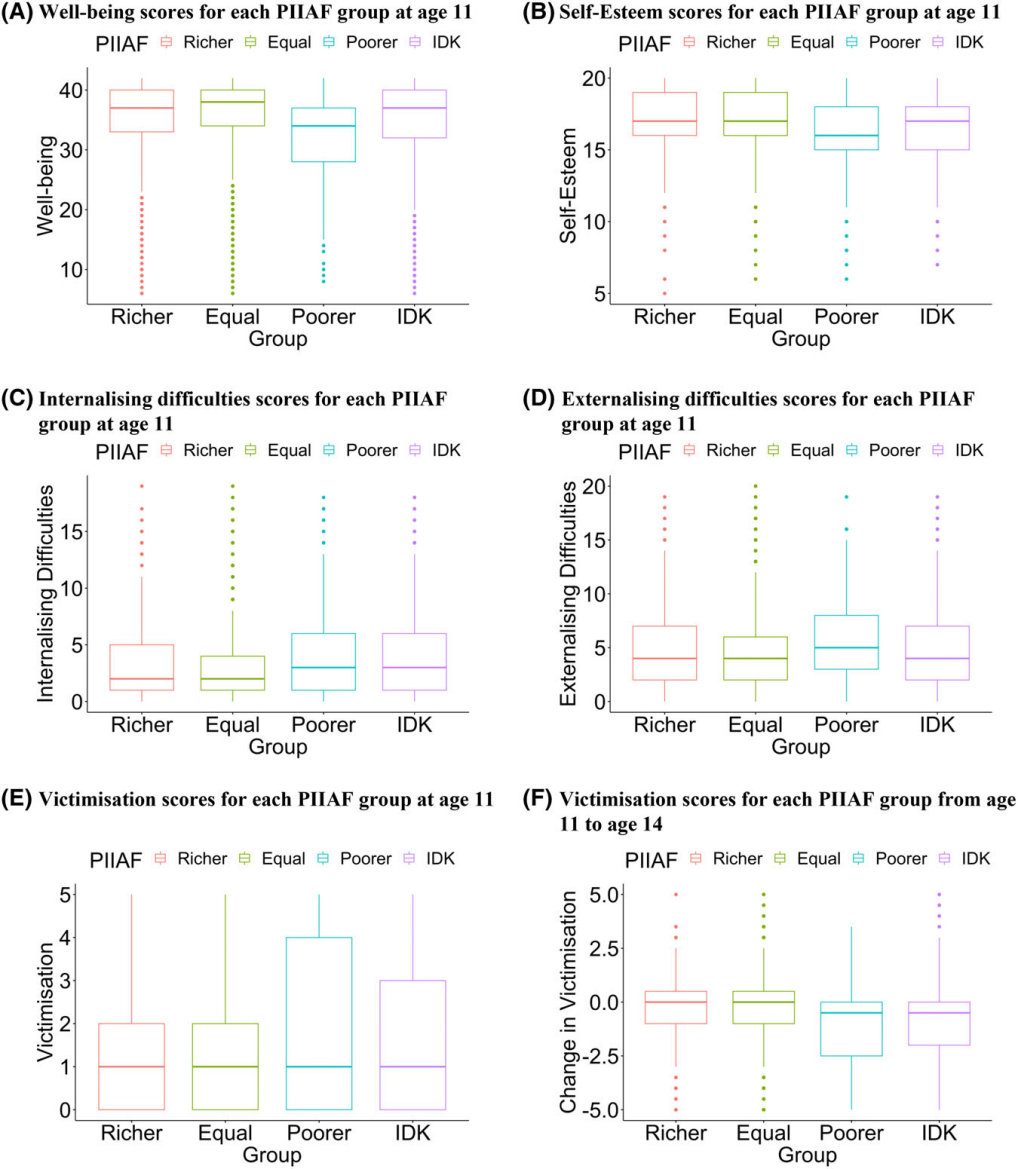
PIIAF group (richer, equal, poorer and I do not know; IDK) differences in each outcome measure at age 11, as well as the change in victimisation from ages 11 to 14, as determined by a set of ANOVAs. *Note*. Each boxplot represents the median score of each PIIAF group and its interquartile range, lines represent the total spread of the data and dots represent outliers for each PIIAF group. Plot A. Poorer PIIAF group shows lower well‐being than all PIIAF groups; the equal PIIAF group shows higher well‐being than all PIIAF groups. Plot B. Poorer PIIAF group shows lower self‐esteem than all PIIAF groups; equal PIIAF and richer PIIAF groups show higher self‐esteem than other PIIAF groups. Plot C. Poorer PIIAF group shows higher internalising difficulties than all other PIIAF groups; equal PIIAF and richer PIIAF groups show lower internalising difficulties than other PIIAF groups. Plot D. Poorer PIIAF group shows higher externalising difficulties than all other PIIAF groups; the equal PIIAF group shows lower externalising difficulties than all PIIAF groups. Plot E. Poorer PIIAF group is victimised more than all PIIAF groups; equal PIIAF group is victimised less than all PIIAF groups. Plot F. Poorer PIIAF group shows a greater decrease in victimisation from ages 11 to 14 than the equal PIIAF and richer PIIAF groups, but not the IDK PIIAF group; equal PIIAF group shows a smaller decrease than the IDK group, but not the richer PIIAF group [Color figure can be viewed at wileyonlinelibrary.com]

In support of hypothesis 1b, post hoc Bonferroni‐corrected comparisons showed that individuals who perceived themselves as poorer than their friends had lower well‐being and self‐esteem scores, higher internalising and externalising difficulties and were victimised more often, than those who perceived themselves as richer, equal and those who did not know (see Figure 2A–E; see Table 3 for relevant comparisons; see Table S5 for all other comparisons). Taking well‐being as an example, adolescents aged 11 who perceive themselves as poorer than their friends had well‐being scores that were 11% lower than adolescents who perceive themselves as the same (a 4‐point difference on a 36‐point scale; see all mean scores of the outcome measures in Table S4).

**Table 3:** Pairwise comparisons between poorer PIIAF and other PIIAF groups resulting from the ANOVAs

Group 1	Group 2	*N* of group 1	*N* of group 2	*t*	*df*	*p* Value	Adjusted *p* Value^a^
Well‐being at age 11							
Equal	Poorer	9,302	527	12.14	560.94	<.001	<.001
IDK	Poorer	2,122	527	7.78	774.65	<.001	<.001
Poorer	Richer	527	1,044	−7.67	1070.34	<.001	<.001
Self‐esteem at age 11							
Equal	Poorer	9,302	527	9.42	526.28	<.001	<.001
IDK	Poorer	2,122	527	5.54	692.95	<.001	<.001
Poorer	Richer	527	1,044	−8.92	876.20	<.001	<.001
Internalising difficulties at age 11							
Equal	Poorer	9,302	527	−7.91	549.46	<.001	<.001
IDK	Poorer	2,122	527	−3.18	750.81	.002	.009
Poorer	Richer	527	1,044	5.85	919.83	<.001	<.001
Externalising difficulties at age 11							
Equal	Poorer	9,302	527	−7.91	556.81	<.001	<.001
IDK	Poorer	2,122	527	−3.49	774.46	.001	.003
Poorer	Richer	527	1,044	4.06	1000.34	<.001	<.001
Bullying at age 11							
Equal	Poorer	9,302	527	−5.24	556.69	<.001	<.001
IDK	Poorer	2,122	527	−3.05	761.72	.002	.014
Poorer	Richer	527	1,044	2.17	1019.84	.030	.179
Victimisation at age 11							
Equal	Poorer	9,302	527	−10.51	556.47	<.001	<.001
IDK	Poorer	2,122	527	−5.80	760.31	<.001	<.001
Poorer	Richer	527	1,044	6.90	960.09	<.001	<.001
Victimisation change scores between ages 11 and 14							
Equal	Poorer	9,302	527	5.80	458.77	<.001	<.001
IDK	Poorer	2,122	527	2.26	621.82	.024	.143
Poorer	Richer	527	1,044	−3.73	799.09	<.001	.001

Pairwise comparisons between poorer PIIAF and other PIIAF groups of ANOVAs conducted on the mean of the outcome scores at age 11 and on the victimisation change scores from ages 11 to 14.

^a^ The adjusted *p* value corresponds to an altered significance level after Bonferroni correction for six multiple comparisons.

Contrary to hypothesis 1b, we found that, while individuals who perceived themselves as poorer reported higher bullying relative to those who perceived themselves as equal and to those who did not, they did not report higher bullying compared to those who perceived themselves as richer (see Table 3 for relevant comparisons; see Table S5 and Figure S2 for all other comparisons).

### Hypothesis 1c

We ran a second set of one‐way ANOVAs to test the hypothesis that individuals who perceived themselves as poorer than their friends at age 11 would report a greater increase in difficulties relative to all other PIIAF groups (i.e. richer, equal and I do not know) from ages 11 to 14 (H1c). An ANOVA showed a small but significant effect of PIIAF on the change of victimisation from ages 11 to 14, *F*(3, 10415) = 30.03, *p* < .001, $\eta_{p}^{2}$ = .01. No other change in outcomes from ages 11 to 14 varied across PIIAF groups, and therefore we did not conduct any further ANOVAs (except binge drinking, see supplemental analyses Appendix SD and Table S6).

Contrary to hypothesis 1c, post hoc Bonferroni‐corrected comparisons showed that individuals who perceived themselves as poorer reported a greater *decrease* in victimisation scores from ages 11 to 14 relative to those who perceived themselves as richer and equal, but not compared to those who did not know (see Figure 2F; see Table 3 for relevant comparisons; see Table S5 for all other comparisons). Note, however, that the correlation between the values at ages 11 and 14 was low (alpha = .44), and therefore, results should be interpreted with caution.

In summary, accounting for PIIAF explained more variance in all outcome measures than only accounting for objective measures of family income, supporting Hypothesis 1a. We further found mixed evidence for Hypothesis 1b, such that those who perceived themselves as belonging to poorer families than their friends' families had more mental health difficulties and were victimised more, but did not bully more, than other PIIAF groups at age 11. Finally, we did not find evidence to support Hypothesis 1c (see Appendix S11 for author's positionality statement, relevant to the interpretation of results).

## Discussion

In the present study, we conducted a preregistered analysis on longitudinal data from the UK MCS to explore the impact of perceived income inequality among friends (PIIAF) on mental health and interpersonal difficulties of young adolescents, controlling for the influence of objective measures of family income. At age 11, cohort members were asked whether they perceived their family as richer, poorer, the same as their friends or whether they did not know. We found that PIIAF explained additional variance in every one of the outcome measures, over and above objective family income.

We examined whether those adolescents who perceived themselves as coming from poorer households than their friends at age 11 fared worse on each of our outcome measures at the same age. We found that young people who perceived themselves as belonging to poorer families than their friends reported worse outcomes for well‐being, self‐esteem, internalising problems, externalising problems and victimisation, relative to those who perceived themselves as richer or equal to their friends, or who did not know. It has been proposed that experiencing both absolute and relative poverty in an environment confers a double disadvantage (e.g. Crosnoe, 2009). This could be especially salient during early adolescence when individuals rely heavily on social information to make judgements about themselves (Odgers, 2015). Our results extend this literature by proposing that economic inequalities are also influential in close relationships, such as friend groups. Therefore, it is important for future research to consider how subjective and objective indices of inequality in more proximal environments (i.e. not country level) can shape perceptions of oneself through comparisons with different reference groups, for example, friends, school peers and neighbourhood peers, young people in the same country and interactions between them (e.g. Douglass, Mirpuri, & Yip, 2017).

In addition, we found that, for bullying perpetration, adolescents who perceived themselves as poorer than their friends reported similar levels of bullying as those who perceived themselves as richer, and both groups reported higher bullying relative to those who perceived themselves as equal. Notably, those who perceived themselves as equal to their friends reported the least difficulties across a number of outcome measures at age 11. In addition to having the lowest rates of bullying perpetration, participants who perceived themselves as equal to their friends also had the lowest externalising problems, were victimised less and had the lowest peer problems in relation to all other PIIAF groups. These findings suggest that for some outcomes, and perhaps especially for those related to interpersonal difficulties, it is not just perceiving one's family as poorer, but rather different (including richer too), to one's friends that is associated with an increased risk for adverse outcomes. This is different from previous literature employing measures of subjective socioeconomic status (SSS), which integrate both economic and social positioning in relation to one's environment, and suggests an inverse relationship between externalising difficulties and SSS (see Highlander & Jones, 2021, for review). Given that belonging is of particular importance to adolescents, one hypothesis is that those who feel economically dissimilar to their friends and peers, independent of their social position within their close environment, might interpret this as a risk to their group belonging, which may increase their risk of experiencing more difficulties related to interpersonal relations (Andrews et al., 2021; Tomova, Andrews, & Blakemore, 2021). While the relationships we find between PIIAF and our outcomes have small effect sizes, and difficulties experienced by individuals across PIIAF will vary on an individual basis, these are still meaningful to understanding health inequalities, as small effects accumulate over populations and over time (Götz, Gosling, & Rentfrow, 2022). In light of this, future work should explore whether decreasing the salience of inequalities in UK school environments could foster belonging in young people (e.g. providing guidance for parents as is common in some Scandinavian cities; see The Norwegian Directorate for Education and Training, 2016).

Finally, we investigated the relationship between PIIAF at age 11 and the change in mental health and interpersonal difficulties from ages 11 to 14. Contrary to expected, we found that victimisation rates decreased for all adolescents from ages 11 to 14, and that these decreased to a greater extent for those who perceived themselves as poorer as opposed to the other PIIAF groups. In addition, we found that the change in the other outcome measures did not differ between PIIAF groups. One possible explanation for these results is that PIIAF might be changing over time for the adolescents included in our sample. Adolescence is a period of social transition when friend and larger peer groups might both be changing constantly. In addition, PIIAF might not perfectly capture changes in inequality, for example, *how much poorer* an individual feels compared to their friends. However, no PIIAF measure was collected at age 14, and thus we were unable to inspect whether PIIAF trajectories differed across groups. A second explanation could be that both PIIAF and our outcome measures are capturing similar constructs (e.g. subjective well‐being). However, we find small standardised estimates across all our age 11 analyses, which suggests that there is variance in all our outcome measures that is not accounted for by PIIAF. Similarly, the relationship between PIIAF and our outcomes might be driven by higher mental health and interpersonal difficulties at age 11 affecting how adolescents perceive themselves at that age. Taken together, these alternative explanations imply that the association among PIIAF, adverse mental health and internalising difficulties at age 11 cannot be fully understood given the limited available measures and timescales for our study. Future longitudinal studies, with more than two waves, should address this limitation by including PIIAF at different time points to better capture PIIAF trajectories across adolescence.

## Conclusion

In summary, we found that at the age of 11, children are sensitive to how wealthy they perceive their family to be compared to their friends, independent of their objective income. Adolescents who perceive their family as poorer are generally at a greater disadvantage than those who perceive their family as equal to their friends. Our results are consistent with previous findings that an uneven distribution of wealth leads to negative outcomes for young people, and further highlight the relevance of considering proximal social environments, including economic inequalities among friend groups. These findings could help direct policy, for example, by seeking to understand how uneven distribution of wealth in schools and other proximal environments (e.g. gentrification; Cole, Mehdipanah, Gullón, & Triguero‐Mas, 2021) presents a risk factor for belonging in young people.

## Rights of use

For the purpose of open access, the author has applied a Creative Commons Attribution (CC BY) licence to any Author Accepted Manuscript version arising from this submission.

**Key points**

- Perceived income inequality among friends (PIIAF) at age 11 accounts for variance in mental health and interpersonal difficulties independently from objective family income.
- Young adolescents (aged 11) who perceive themselves as belonging to poorer families than their friends experience worse mental health difficulties and are victimised more than those who do not perceive themselves this way.
- Young adolescents (aged 11) who perceive themselves as belonging to families equal in wealth to their friends experience the best outcomes.
- Future longitudinal research should investigate how PIIAF trajectories are related to the salience of economic inequalities in proximal social environments and their relationship to belonging in young people.

## Supporting information


**Appendix S1**. Power simulations.
**Appendix S2**. Well‐being questions.
**Appendix S3**. Self‐esteem questions.
**Appendix S4**. Strengths and difficulties questions.
**Appendix S5**. Bullying and victimisation questions.
**Appendix S6**. Drinking and binge drinking questions.
**Appendix S7**. MCS specified survey UK‐wide weights.
**Appendix S8**. Objective family income.
**Appendix S9**. Preregistered Exploratory Model Comparisons.
**Appendix S10**. Best fitting model for each outcome measure.
**Appendix S11**. Author Positionality Statement.
**Appendix SA.** Emotional difficulties.
**Appendix SB.** Peer problems.
**Appendix SC.** Drinking behaviour.
**Appendix SD.** Binge drinking behaviour.
**Figure S1.** EFI per PIIAF group.
**Figure S2.** PIIAF group (richer, equal, poorer and I do not know; IDK) differences in bullying at age 11 as determined by an ANOVA.
**Table S1.** Results for χ^2^ and BIC difference tests for each outcome measure after controlling for sample weights.
**Table S2.** Results for χ^2^ and BIC difference tests for each outcome measure after controlling for objective family income, as measured by country‐level quantiles of EFI.
**Table S3.** Model fit statistics for each outcome measure.
**Table S4.** Mean scores for each outcome measure when cohort members are age 11 and age 14, grouped by PIIAF (equal, richer, poorer and IDK).
**Table S5.** Pairwise comparisons resulting from each one‐way ANOVA.
**Table S6.** Pairwise comparisons resulting from emotional difficulties, peer problems, drinking and binge drinking one‐way ANOVAs.

## References

[jcpp13719-bib-0001] Andrews, J.L. , Ahmed, S.P. , & Blakemore, S.J. (2021). Navigating the social environment in adolescence: The role of social brain development. Biological Psychiatry, 89, 109–118. 33190844 10.1016/j.biopsych.2020.09.012

[jcpp13719-bib-0002] Braveman, P. , Egerter, S. , & Williams, D.R. (2011). The social determinants of health: Coming of age. Annual Review of Public Health, 32, 381–398. 10.1146/annurev-publhealth-031210-101218 21091195

[jcpp13719-bib-0003] Cole, H.V. , Mehdipanah, R. , Gullón, P. , & Triguero‐Mas, M. (2021). Breaking down and building up: Gentrification, its drivers, and urban health inequality. Current Environmental Health Reports, 8, 157–166. 33713334 10.1007/s40572-021-00309-5 PMC7955692

[jcpp13719-bib-0004] Connelly, R. , & Platt, L. (2014). Cohort Profile: UK Millennium Cohort Study (MCS). International Journal of Epidemiology, 43, 1719–1725. 24550246 10.1093/ije/dyu001

[jcpp13719-bib-0005] Crosnoe, R. (2009). Low‐income students and the socioeconomic composition of public high schools. American Sociological Review, 74, 709–730. 21546987 10.1177/000312240907400502 PMC3086272

[jcpp13719-bib-0006] Dalmaijer, E.S. , Gibbons, S.G. , Bignardi, G. , Anwyl‐Irvine, A.L. , Siugzdaite, R. , Smith, T.A. , … & Astle, D.E. (2021). Direct and indirect links between children's socio‐economic status and education: Pathways via mental health, attitude, and cognition. Current Psychology, 1–15. 10.1007/s12144-021-02232-2 PMC7614555 37215737

[jcpp13719-bib-0007] Douglass, S. , Mirpuri, S. , & Yip, T. (2017). Considering friends within the context of peers in school for the development of ethnic/racial identity. Journal of Youth and Adolescence, 46, 300–316. 27464751 10.1007/s10964-016-0532-0 PMC6055928

[jcpp13719-bib-0008] Elgar, F.J. , Gariepy, G. , Dirks, M. , Walsh, S.D. , Molcho, M. , Cosma, A. , … & Craig, W. (2019). Association of early‐life exposure to income inequality with bullying in adolescence in 40 countries. JAMA Pediatrics, 173, e191181. 31081857 10.1001/jamapediatrics.2019.1181 PMC6515581

[jcpp13719-bib-0009] Elgar, F.J. , Pförtner, T.K. , Moor, I. , De Clercq, B. , Stevens, G.W. , & Currie, C. (2015). Socioeconomic inequalities in adolescent health 2002–2010: A time‐series analysis of 34 countries participating in the health behaviour in school‐aged children study. The Lancet, 385, 2088–2095. 10.1016/S0140-6736(14)61460-4 25659283

[jcpp13719-bib-0010] Goodman, A. , & Goodman, R. (2009). Strengths and difficulties questionnaire as a dimensional measure of child mental health. Journal of the American Academy of Child & Adolescent Psychiatry, 48, 400–403. 19242383 10.1097/CHI.0b013e3181985068

[jcpp13719-bib-0011] Götz, F.M. , Gosling, S.D. , & Rentfrow, P.J. (2022). Small effects: The indispensable foundation for a cumulative psychological science. Perspectives on Psychological Science, 17, 205–215. 34213378 10.1177/1745691620984483

[jcpp13719-bib-0012] Highlander, A.R. , & Jones, D.J. (2021). Integrating objective and subjective social class to advance our understanding of externalizing problem behavior in children and adolescents: A conceptual review and model. Clinical Child and Family Psychology Review, 25, 300–315. 34533656 10.1007/s10567-021-00369-x PMC11907708

[jcpp13719-bib-0013] Kersten, P. , Czuba, K. , McPherson, K. , Dudley, M. , Elder, H. , Tauroa, R. , & Vandal, A. (2016). A systematic review of evidence for the psychometric properties of the strengths and difficulties questionnaire. International Journal of Behavioral Development, 40, 64–75.

[jcpp13719-bib-0014] Kessler, R.C. , Amminger, G.P. , Aguilar‐Gaxiola, S. , Alonso, J. , Lee, S. , & Ustun, T.B. (2007). Age of onset of mental disorders: A review of recent literature. Current Opinion in Psychiatry, 20, 359–364. 17551351 10.1097/YCO.0b013e32816ebc8c PMC1925038

[jcpp13719-bib-0015] Kievit, R.A. , Brandmaier, A.M. , Ziegler, G. , Van Harmelen, A.L. , De Mooij, S.M. , Moutoussis, M. , … & NSPN Consortium . (2018). Developmental cognitive neuroscience using latent change score models: A tutorial and applications. Developmental Cognitive Neuroscience, 33, 99–117. 29325701 10.1016/j.dcn.2017.11.007 PMC6614039

[jcpp13719-bib-0016] Kline, R.B. (2015). Principles and practice of structural equation modeling. London, UK: Guilford.

[jcpp13719-bib-0017] Lynch, J.W. , Kaplan, G.A. , & Shema, S.J. (1997). Cumulative impact of sustained economic hardship on physical, cognitive, psychological, and social functioning. New England Journal of Medicine, 337, 1889–1895. 9407157 10.1056/NEJM199712253372606

[jcpp13719-bib-0018] McArdle, J.J. (2009). Latent variable modeling of differences and changes with longitudinal data. Annual Review of Psychology, 60, 577–605. 10.1146/annurev.psych.60.110707.163612 18817479

[jcpp13719-bib-0019] McLaughlin, K.A. , Costello, E.J. , Leblanc, W. , Sampson, N.A. , & Kessler, R.C. (2012). Socioeconomic status and adolescent mental disorders. American Journal of Public Health, 102, 1742–1750. 22873479 10.2105/AJPH.2011.300477 PMC3482020

[jcpp13719-bib-0020] Murray, A.L. , Speyer, L.G. , Hall, H.A. , Valdebenito, S. , & Hughes, C. (2022). A longitudinal and gender invariance analysis of the strengths and difficulties questionnaire across ages 3, 5, 7, 11, 14, and 17 in a large U.K.‐representative sample. Assessment, 29(6), 1248–1261. 33874786 10.1177/10731911211009312 PMC9301174

[jcpp13719-bib-0021] Odgers, C.L. (2015). Income inequality and the developing child: Is it all relative? American Psychologist, 70, 722–731. 26618957 10.1037/a0039836 PMC4784260

[jcpp13719-bib-0022] Odgers, C.L. , Donley, S. , Caspi, A. , Bates, C.J. , & Moffitt, T.E. (2015). Living alongside more affluent neighbors predicts greater involvement in antisocial behavior among low‐income boys. Journal of Child Psychology and Psychiatry, 56, 1055–1064. 25611118 10.1111/jcpp.12380 PMC4790437

[jcpp13719-bib-0023] Pickett, K.E. , & Wilkinson, R.G. (2007). Child wellbeing and income inequality in rich societies: Ecological cross sectional study. BMJ, 335, 1080. 18024483 10.1136/bmj.39377.580162.55 PMC2094139

[jcpp13719-bib-0024] Pickett, K.E. , & Wilkinson, R.G. (2010). Inequality: An underacknowledged source of mental illness and distress. The British Journal of Psychiatry, 197, 426–428. 21119145 10.1192/bjp.bp.109.072066

[jcpp13719-bib-0025] Quon, E.C. , & McGrath, J.J. (2014). Subjective socioeconomic status and adolescent health: A meta‐analysis. Health Psychology, 33, 433–447. 24245837 10.1037/a0033716 PMC5756083

[jcpp13719-bib-0026] Rivenbark, J. , Arseneault, L. , Caspi, A. , Danese, A. , Fisher, H.L. , Moffitt, T.E. , … & Odgers, C.L. (2020). Adolescents' perceptions of family social status correlate with health and life chances: A twin difference longitudinal cohort study. Proceedings of the National Academy of Sciences of the United States of America, 117, 23323–23328. 31907315 10.1073/pnas.1820845116 PMC7519389

[jcpp13719-bib-0027] Robins, R.W. , Hendin, H.M. , & Trzesniewski, K.H. (2001). Measuring global self‐esteem: Construct validation of a single‐item measure and the Rosenberg self‐esteem scale. Personality and Social Psychology Bulletin, 27, 151–161.

[jcpp13719-bib-0028] Rosseel, Y. (2012). Lavaan: An R package for structural equation modeling. Journal of Statistical Software, 48, 1–36.

[jcpp13719-bib-0029] Satorra, A. , & Bentler, P.M. (2010). Ensuring positiveness of the scaled difference chi‐square test statistic. Psychometrika, 75, 243–248. 20640194 10.1007/s11336-009-9135-y PMC2905175

[jcpp13719-bib-0030] Sawyer, S.M. , Azzopardi, P.S. , Wickremarathne, D. , & Patton, G.C. (2018). The age of adolescence. The Lancet Child & Adolescent Health, 2, 223–228. 30169257 10.1016/S2352-4642(18)30022-1

[jcpp13719-bib-0031] Schermelleh‐Engel, K. , Moosbrugger, H. , & Muller, H. (2003). Evaluating the fit of structural equation models: Tests of significance and descriptive goodness‐of‐fit measures. Methods of Psychological Research Online, 8, 23–74.

[jcpp13719-bib-0032] Scott, K.M. , Al‐Hamzawi, A.O. , Andrade, L.H. , Borges, G. , Caldas‐de‐Almeida, J.M. , Fiestas, F. , … & Kessler, R.C. (2014). Associations between subjective social status and DSM‐IV mental disorders: Results from the world mental health surveys. JAMA Psychiatry, 71, 1400–1408. 25354080 10.1001/jamapsychiatry.2014.1337 PMC5315238

[jcpp13719-bib-0033] Shepherd, P. , & Gilbert, E. (2019). Millennium cohort study ethical review and consent (2nd edn). London, UK: Center for Longitudinal Studies. www.cls.ucl.ac.uk

[jcpp13719-bib-0034] Somerville, L.H. (2013). The teenage brain: Sensitivity to social evaluation. Current Directions in Psychological Science, 22, 121–127. 24761055 10.1177/0963721413476512 PMC3992953

[jcpp13719-bib-0035] The Norwegian Directorate for Education and Training . (2016). “Vaksne skaper vennskap”. https://www.udir.no/laring‐ogtrivsel/mobbing/voksne‐skaper‐vennskap/

[jcpp13719-bib-0036] Tomova, L. , Andrews, J.L. , & Blakemore, S.J. (2021). The importance of belonging and the avoidance of social risk taking in adolescence. Developmental Review, 61, 100981.

[jcpp13719-bib-0037] Van der Aar, L.P.E. , Peters, S. , & Crone, E.A. (2018). The development of self‐views across adolescence: Investigating self‐descriptions with and without social comparison using a novel experimental paradigm. Cognitive Development, 48, 256–270.

