# Supplementary material for: The relationship between perceived income inequality, adverse mental health and interpersonal difficulties in UK adolescents
Source: J Child Psychol Psychiatry. 2022 Nov 14;64(3):417–25. doi: 10.1111/jcpp.13719 (PMC10100326; doi:10.1111/jcpp.13719)
Supplement: Supplementary file 1 — Appendix S1. Power simulations. Appendix S2. Well‐being questions. Appendix S3. Self‐esteem questions. Appendix S4. Strengths and difficulties questions. Appendix S5. Bullying and victimisation questions. Appendix S6. Drinking and binge drinking questions. Appendix S7. MCS specified survey UK‐wide weights. Appendix S8. Objective family income. Appendix S9. Preregistered Exploratory Model Comparisons. Appendix S10. Best fitting model for each outcome measure. Appendix S11. Author Positionality Statement. Appendix SA. Emotional difficulties. Appendix SB. Peer problems. Appendix SC. Drinking behaviour. Appendix SD. Binge drinking behaviour. Figure S1. EFI per PIIAF group. Figure S2. PIIAF group (richer, equal, poorer and I do not know; IDK) differences in bullying at age 11 as determined by an ANOVA. Table S1. Results for χ2 and BIC difference tests for each outcome measure after controlling for sample weights. Table S2. Results for χ2 and BIC difference tests for each outcome measure after controlling for objective family income, as measured by country‐level quantiles of EFI. Table S3. Model fit statistics for each outcome measure. Table S4. Mean scores for each outcome measure when cohort members are age 11 and age 14, grouped by PIIAF (equal, richer, poorer and IDK). Table S5. Pairwise comparisons resulting from each one‐way ANOVA. Table S6. Pairwise comparisons resulting from emotional difficulties, peer problems, drinking and binge drinking one‐way ANOVAs. [file JCPP-64-417-s001.docx]

**Supporting Information**

**Appendix S1. Power Simulations**

Given the unbalanced grouping of the PIIAF measure (Equal *n=*9302; IDK *n=*2102; Richer n=1044; Poorer *n=*527), we ran a set of *a posteriori* power simulations upon reviewer request.

Specifically, we ran 3 separate simulations:

1. We used the self-esteem LCSM parameter estimates to simulate 500 unbalanced datasets using the R package *simsem* (which uses *lavaan* output). We then compared a model that freed the five parameters to vary across PIIAF to a model which constrained the parameters to be equal across PIIAF. We found that these simulated datasets had >99% power to detect a significant (alpha = .05) difference between the freed and the constrained model.
2. We used the descriptive means and standard deviations of the self-esteem data to simulate 500 unbalanced datasets. We then ran a one-way ANOVA in each dataset and found that these simulated datasets had >99% power to detect a significant (alpha = .05) effect of PIIAF on the simulated data.
3. We used the descriptive means and standard deviations of the self-esteem data to simulate 500 unbalanced datasets. We then ran a set of Bonferroni corrected post-hoc comparisons and found that these simulated datasets had >99% power to detect a significant (alpha = .05) difference between Poorer PIIAF and the other PIIAF groups.

**Appendix S2. Well-being questions**

The questions below are taken from the young person questionnaire completed by participants at age 11. About 2.4% of the participants had missing data for well-being at age 11 and 19.7% at age 14.

**Appendix S3. Self-esteem questions**

The questions below are taken from the young person questionnaire completed by participants at age 11. About 6.5% of participants had missing data for self-esteem at age 11, and 20.2% at age 14.

**Appendix S4. Strengths and difficulties questions**

The scoring sheet below is taken from the Youth in Mind website (https://www.sdqinfo.org/) and includes the parental report questions for each of the strengths and difficulties subscales. The emotional difficulties and the peer problems subscales are aggregated to get a measure of internalising difficulties, and the hyperactivity and the conduct problems scale are aggregated to get a measure of externalising difficulties. About 4.4% of the participants had missing data for the internalising difficulties scores at age 11, and 18.7% at age 14, and 4.6% participants had missing data for externalising difficulties at age 11, and 18.7% at age 14.

**

**Appendix S5. Bullying and victimisation questions**

The questions below are taken from the young person questionnaire completed by participants at age 14. At age 11, participants are only asked the first two questions. About 0.01% of the participants had missing data at age 11, and 19.2% at age 14, for both victimisation and behaviour.

**Appendix S6. Drinking and binge drinking questions**

The questions below are taken from the young person questionnaire completed by participants at age 11. About 2.1% of the participants had missing data for drinking behaviour at age 11, and 21.7% at age 14. About 2.1% of the participants had missing data for binge drinking behaviour at age 11, and 19.2% in at age 14.

**Appendix S7. MCS specified survey UK-wide weights**

The MSC sample was clustered, geographically, and disproportionately stratified to over-represent areas with high proportions of ethnic minorities in England, residents of areas of high child poverty and residents of the three smaller countries of the UK respectively. The sample design weights, or probability weights can be used to correct for MCS cases having unequal probabilities of selection that result from the stratified cluster sample design (for more information see MCS Sweep 1 – 5 user guide). To control for this, we used *lavaan.survey* to set the stratified cluster sample design to *weight2*, which is a variable containing the UK-wide sample weights. Table S1 shows that a model additionally including the PIIAF variance also explained variance in all but one (i.e., emotional difficulties) of our outcome measures better than a control model when including the sample weights.

**Appendix S8. Objective family income**

The MCS dataset includes four measures of objective family income derived from the OCED equalised income scale: (1) a continuous measure of net equalised weekly family income (EFI) which divides the total net income by the number of household members according to their weight on the OCED scale; (2) an ordinal transformation of EFI into quintiles based upon UK income distribution; (3) an ordinal transformation of EFI into quintiles based upon income distribution within respondent’s country; and (4) a binary transformation of EFI that flags whether the respondent is above or below 60% of the median income level. We pre-registered and used the continuous measure of EFI (1) as a measure of objective family income for our control models in all our analyses, which converges with PIIAF (see Figure S1). However, we also ran supplemental analyses to check that adolescents’ perceived income inequality (relative to peers; PIIAF) explained differences in mental health and interpersonal difficulties, above any differences explained by objective family income (H1), using the country-specific quantiles of EFI (3). Table S1 shows that a model additionally including the PIIAF variance also explained variance in all our outcome measures better than a control model when using country-specific quantiles of EFI as a measure of objective family income (instead of continuous UK-wide EFI). Table S2 shows *χ^2^* model comparisons for each outcome measure between a model including Gender, Ethnicity and Country-Specific Quantiles of EFI, and a model additionally including PIIAF. The models additionally including PIIAF fit best for all outcome measures.

*Figure S1*

EFI per PIIAF group


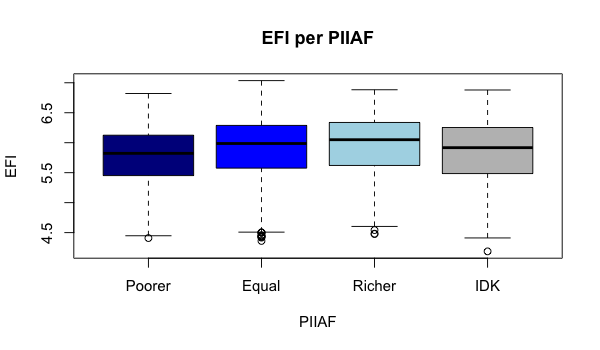


**Appendix S9. Preregistered Exploratory Model Comparisons**

If, for a certain outcome variable, hypothesis 1 was supported, we tested a series of exploratory model comparisons assessing whether freeing each of the five parameters to vary by PIIAF would improve model fit. This allowed us to examine how specifically PIIAF influenced the outcome variable over time and helped determine which parameters would be relevant to test hypotheses 1a and 1b.

To do so, we took a fully constrained model, i.e., a model that constrained all five parameters to be equal across levels of PIIAF, and compared it with a model that freed a certain parameter. If the model that freed the parameter fit better, we continued with this parameter being freed in the next test and then examined whether freeing another parameter improved model fit. We tested the parameters in the following order: mean at age 11 *μDV_i,11_*, mean of the change score *μΔDV_i_*, variance at age 11 *σ^2^DV_i,11_*, variance of the change score *σ^2^ΔDV_i_* and the self-feedback parameter β. We only tested hypothesis 1a on outcome measures where the parameters for the mean at age 11 *μDV_i,11_*, and hypothesis 1b on outcome measures where the mean of the change score *μΔDV_i_* varied freely with PIIAF.

**Appendix S10. Best fitting model for each outcome measure.**

***Well-being***

The best fitting model for well-being is one that freed the intercept at age 11 (Δ*χ^2^* = 195.25, Δdf = 3, *p* < .001, ΔBIC = 197) but constrained the variance at age 11 (Δ*χ^2^* = 26.84, Δdf = 3, *p* < .001, ΔBIC = 7), constrained the change score and the variance from age 11 to age 14 (change: Δ*χ^2^* = 5.44, Δdf = 3, *p* = .142, ΔBIC = -23; variance: Δ*χ^2^* = 7.11, Δdf = 3, *p* = .069, ΔBIC = -18), and constrained the self-regressive parameter (Δ*χ^2^* = 10.49, Δdf = 3, *p* = .015, ΔBIC = -16).

***Self-esteem***

The best fitting model for self-esteem is one that freed the intercept (Δ*χ^2^* = 171.04, Δdf = 3, *p* < .001, ΔBIC = 171) and the variance (Δ*χ^2^* = 56.53, Δdf = 3, *p* < .001, ΔBIC = 41) at age 11, constrained the change score and the variance from age 11 to age 14 (change: Δ*χ^2^* = 3.90, Δdf = 3, *p* = .273, ΔBIC = -24; variance: Δ*χ^2^* =8.05, Δdf = 3, *p* = .045, ΔBIC = -17) and constrained the self-regressive parameter (Δ*χ^2^* = 12.42, Δdf = 3, *p* = .006, ΔBIC = -13).

***Internalising problems***

The best fitting model for internalising difficulties is one that freed the intercept (Δ*χ^2^* = 110.71, Δdf = 3, *p* < .001, ΔBIC = 104), and the variance (Δ*χ^2^* = 47.23, Δdf = 3, *p* < .001, ΔBIC = 54) at age 11, constrained the change score and the variance from age 11 to age 14 (change: Δ*χ^2^* = 4.99, Δdf = 3, *p* = .173, ΔBIC = -23; variance: Δ*χ^2^* = 11.52, Δdf = 3, *p* = .009, ΔBIC = -8), and constrained the self-regressive parameter (Δ*χ^2^* = 4.33, Δdf = 3, *p* = .228, ΔBIC = -22).

***Externalising problems***

The best fitting model for externalising difficulties is one that freed the intercept (Δ*χ^2^* = 72.62, Δdf = 3, *p* < .001, ΔBIC = 55) and the variance (Δ*χ^2^* = 33.67, Δdf = 3, *p* < .001, ΔBIC = 12) at age 11, constrained the change score and the variance from age 11 to age 14 (change: Δ*χ^2^* = 7.35, Δdf = 3, *p* = .062, ΔBIC = -21; variance: Δ*χ^2^* = 3.92, Δdf = 3, *p* = .349, ΔBIC = -23), and additionally constrained the self-regressive parameter (Δ*χ^2^* = 3.81, Δdf = 3, *p* = .293, ΔBIC = -25).

***Bullying***

The best fitting model for bullying was one that freed the intercept at age 11 (Δ*χ^2^* = 56.63, Δdf = 3, *p* < .001, ΔBIC = 43), constrained the change score from age 11 to age 14 (Δ*χ^2^* = 4.84, Δdf = 3, *p* = .184, ΔBIC = -23), freed the variance of the intercept at age 11, as well as the variance of the change score from age 11 to age 14 (Δ*χ^2^* = 43.27, Δdf = 3, *p* < .001, ΔBIC = 122; and Δ*χ^2^* = 12.29, Δdf = 3, *p* = .006, ΔBIC = 15, respectively), and additionally freed the self-regressive parameter (Δ*χ^2^* = 39.74, Δdf = 3, *p* < .001, ΔBIC = 105).

***Victimisation***

Th best fitting model for victimisation was one that freed the intercept (Δ*χ^2^* = 123.72, Δdf = 3, *p* < .001, ΔBIC = 115) and the variance (Δ*χ^2^* = 42.48, Δdf = 3, *p* < .001, ΔBIC = 35) at age 11, freed the change score (Δ*χ^2^* = 60.85, Δdf = 3, *p* < .001, ΔBIC = 46) and the variance (Δ*χ^2^* = 47.11, Δdf = 6, *p* < .001, ΔBIC = 13) from age 11 to age 14, and additionally freed the self-regressive parameter (Δ*χ^2^* = 131.59, Δdf = 3, *p* < .001, ΔBIC = 62).

***Emotional Difficulties***

The best fitting model for emotional difficulties was one that freed the mean at age 11 (Δ*χ^2^* = 65.57, Δdf = 3, *p* < .001, ΔBIC = 45), constrained the mean of the change score from age 11 to age 14 (Δ*χ^2^* = 3.51, Δdf = 3, *p* = .319, ΔBIC = -25), constrained variance at age 11, as well as the variance of the change score from age 11 to age 14 (Δ*χ^2^* = 21.07, Δdf = 3, *p* < .001, ΔBIC = 4; and Δ*χ^2^* = 12.12, Δdf = 3, *p* = .007, ΔBIC = -9, respectively), and constrained the self-regressive parameter (Δ*χ^2^* = 2.45, Δdf = 3, *p* = .484, ΔBIC = -25).

***Peer Problems***

The best fitting model for peer problems was one that freed the mean (Δ*χ^2^* = 108.85, Δdf = 3, *p* < .001, ΔBIC = 105) and the variance (Δ*χ^2^* = 79.15, Δdf = 3, *p* < .001, ΔBIC = 112) at age 11, constrained the mean and the variance of the change score from age 11 to age 14 (change: Δ*χ^2^* = 6.31, Δdf = 3, *p* = .098, ΔBIC = -22; variance: Δ*χ^2^* = 19.71, Δdf = 3, *p* < .001, ΔBIC = 3), and constrained the self-regressive parameter (Δ*χ^2^* = 8.97, Δdf = 3, *p* = .030, ΔBIC = -16).

***Drinking Behaviour***

The best fitting model for drinking behaviour was one that freed the intercept (Δ*χ^2^* = 21.8, Δdf = 3, *p* < .001, ΔBIC = 11), constrained the change score from age 11 to age 14 (Δ*χ^2^* = 14.94, Δdf = 3, *p* = .002, ΔBIC = -9), freed the variance of the intercept at age 11, as well as the variance of the change score from age 11 to age 14 (Δ*χ^2^* = 27.46, Δdf = 3, *p* < .001, ΔBIC = 2071; and Δ*χ^2^* = 15.09, Δdf = 3, *p* = .002, ΔBIC = 77, respectively), and additionally freed the self-regressive parameter (Δ*χ^2^* = 26.03, Δdf = 3, *p* < .001, ΔBIC = 152).

***Binge Drinking Behaviour***

The best fitting model for binge drinking behaviour is one that constrained the intercept at age 11 (Δ*χ^2^* = 11.87, Δdf = 3, *p* = .008, ΔBIC = -8), frees the variance at age 11 (Δ*χ^2^* = 8.81, Δdf = 3, *p* = .032, ΔBIC = 707) at age 11, freed the change score (Δ*χ^2^* = 32.54, Δdf = 3, *p* < .001, ΔBIC = 11) and its variance (Δ*χ^2^* = 24.83, Δdf = 3, *p* < .001, ΔBIC = 226) from age 11 to age 14, and constrained the self-regressive parameter (Δ*χ^2^* = 2.76, Δdf = 3, *p* = .431, ΔBIC = -1).

**Appendix S11. Author Positionality Statement**

I am aware of the roles and responsibilities that stem from my positionality. In the context of this study, while I lack understanding of the direct experience of absolute and relative poverty, I participate in a society whose inequalities are high, and shape the way vulnerabilities are socially reproduced. I therefore use my positionality to contribute to the body of research that aims to inform policy on the negative consequences affecting young people facing social inequality.

**Supplemental Analyses**

**Appendix SA. Emotional Difficulties**

The likelihood ratio test suggested that accounting for PIIAF explained emotional difficulties better than a control model without this grouping variable (Δ*χ^2^* = 106.07, Δdf = 15, *p* < .001, ΔBIC = 47), giving support to Hypothesis 1.

A one-way ANOVAs showed a small but significant effect of PIIAF on all emotional difficulties at age 11 (*F*(3,12426)=29.09, *p* < .001, *η_p_^2^*=.007). Post hoc Bonferroni corrected comparisons found that the individuals who perceived themselves as poorer had higher emotional difficulties relative to those who perceived themselves as richer (*p* < .001), equal (*p* < .001), and those who did not know (*p* = .005; see Table S6 for all comparisons).

**Appendix SB. Peer Problems**

The likelihood ratio test suggested that accounting for PIIAF explained peer problems better than a control model without this grouping variable (Δ*χ^2^* = 244.6, Δdf = 15, *p* < .001, ΔBIC = 254), giving support to Hypothesis 1.

A one-way ANOVAs showed a small but significant effect of PIIAF on all peer problems at age 11 (*F*(3,12432)=55.71, *p* < .001, *η_p_^2^*=.013). Post-hoc Bonferroni corrected comparisons found that the individuals who perceived themselves as poorer had higher peer problems relative to those who perceived themselves as richer (*p* < .001), equal (*p* < .001), but not those who did not know (*p* =.211; see Table S6 for all comparisons).

**Appendix SC. Drinking behaviour**

The likelihood ratio test suggested that accounting for PIIAF explained drinking behaviour better than a control model without this grouping variable (Δ*χ^2^* = 128.5, Δdf = 15, *p* < .001, ΔBIC = 1585), giving support to Hypothesis 1.

A one-way ANOVAs showed a small but significant effect of PIIAF on all drinking behaviour at age 11 (*F*(3, 12718)=10.33, *p* < .001, *η_p_^2^*=.002). Post-hoc Bonferroni corrected comparisons found no differences between PIIAF groups (see Table S6 for all comparisons).

**Appendix SD. Binge drinking behaviour**

The likelihood ratio test suggested that accounting for PIIAF explained drinking behaviour better than a control model without this grouping variable (Δ*χ^2^* = 50.36, Δdf = 15, *p* < .001, ΔBIC = 969), giving support to Hypothesis 1.

A one-way ANOVAs showed a small but significant main effect of PRI on change in binge drinking behaviour from age 11 to age 14 (*F*(3,10297)=9.43, *p* < .001, *η_p_^2^*=.003). Post-hoc Bonferroni corrected comparisons found that individuals who perceived themselves as poorer reported a greater increase in binge drinking behaviour from age 11 to age 14 relative to those who did not know only, but not compared to those who perceived themselves richer or equal (see Table S6 for all comparisons).

**Supplemental Tables**

**Table S1**

| ***Outcome measure*** | ***Δχ^2^*** | ***ΔDf*** | ***p-value*** | ***ΔBIC*** |
| --- | --- | --- | --- | --- |
| *Well-being* | 278.3 | 15 | <.001 | 136 |
| *Self-Esteem* | 273.19 | 15 | <.001 | 131 |
| *Internalising Difficulties* | 235.9 | 15 | <.001 | 94 |
| *Externalising Difficulties* | 122.87 | 15 | <.001 | 93 |
| *Bullying Behaviour* | 373.21 | 15 | <.001 | 231 |
| *Being Victimised* | 372.76 | 15 | <.001 | 231 |
| *Drinking Behaviour* | 1374.3 | 15 | <.001 | 1232 |
| *Binge Drinking Behaviour* | 840.65 | 15 | <.001 | 698 |
| *Emotional Difficulties* | 95.03 | 15 | <.001 | -47 |
| *Peer Problems* | 402.45 | 15 | <.001 | 260 |

*Results for χ^2^ and BIC difference tests for each outcome measure after controlling for sample weights*

**Table S2**

*Results for χ^2^ and BIC difference tests for each outcome measure after controlling for objective family income, as measured by country-level quantiles of EFI*

| ***Outcome measure*** | ***Δχ^2^*** | ***ΔDf*** | ***p-value*** | ***ΔBIC*** |
| --- | --- | --- | --- | --- |
| *Well-being* | 191.56 | 15 | *p* < .001 | 187 |
| *Self-Esteem* | 258.08 | 15 | *p* < .001 | 221 |
| *Internalising Difficulties* | 184.12 | 15 | *p* < .001 | 170 |
| *Externalising Difficulties* | 110.47 | 15 | *p* < .001 | 44 |
| *Bullying Behaviour* | 159.8 | 15 | *p* < .001 | 309 |
| *Being Victimised* | 311 | 15 | *p* < .001 | 281 |
| *Emotional Difficulties* | 105.47 | 15 | *p* < .001 | 46 |
| *Peer Problems* | 245.06 | 15 | *p* < .001 | 253 |
| *Drinking Behaviour* | 128.16 | 15 | *p* < .001 | 1584 |
| *Binge Drinking Behaviour* | 50.27 | 15 | *p* < .001 | 966 |

**Table S3**

*Model fit statistics for each outcome measure*

| ***Outcome measure*** | ***CFI*** | ***RMSEA*** | ***RMSEA UPPER CI*** | ***RMSEA LOWER CI*** | ***SRMR*** | ***TLI*** |
| --- | --- | --- | --- | --- | --- | --- |
| *Well-being* | .986 | .037 | .040 | .034 | .035 | .981 |
| *Self-Esteem* | .987 | .036 | .039 | .033 | .034 | .982 |
| *Internalising Difficulties* | .988 | .037 | .040 | .034 | .034 | .983 |
| *Externalising Difficulties* | .989 | .037 | .040 | .034 | .035 | .985 |
| *Bullying Behaviour* | .986 | .037 | .040 | .034 | .034 | .981 |
| *Being Victimised* | .987 | .037 | .040 | .034 | .034 | .982 |
| *Emotional Difficulties* | .987 | .037 | .040 | .034 | .034 | .983 |
| *Peer Problems* | .988 | .036 | .039 | .033 | .034 | .983 |
| *Drinking Behaviour* | .987 | .036 | .039 | .033 | .034 | .982 |
| *Binge Drinking Behaviour* | .987 | .035 | .038 | .032 | .034 | .982 |

**Table S4**

*Mean scores for each outcome measure when cohort members are age 11 and age 14, grouped by PIIAF (equal, richer, poorer and IDK)*

| ***Outcome measure*** | ***Equal PIIAF***  ***(n = 9302)*** | ***Richer PIIAF***  ***(n = 1044)*** | ***Poorer PIIAF***  ***(n = 527)*** | ***IDK PIIAF***  ***(n = 2122)*** |
| --- | --- | --- | --- | --- |
| **Well-being** | | | | |
| range age 11 | 6 – 42 | 6– 42 | 8 – 42 | 6 – 42 |
| mean age 11 (sd) | 36.07 (6.19) | 35.24 (6.97) | 32.43 (6.63) | 34.97 (6.50) |
| range age 14 | 6 – 42 | 6 – 42 | 9 – 42 | 6 – 42 |
| mean age 14 (sd) | 33.26 (6.48) | 33.02 (6.78) | 30.28 (7.38) | 32.71 (6.71) |
| **Self-Esteem** | | | | |
| range age 11 | 6 – 20 | 6– 20 | 6 – 20 | 7 – 20 |
| mean age 11 (sd) | 17.06 (2.07) | 17.17 (2.29) | 15.92 (2.65) | 16.64 (2.28) |
| range age 14 | 5 – 20 | 5 – 20 | 5 – 20 | 5 – 20 |
| mean age 14 (sd) | 15.64 (2.85) | 15.78 (2.94) | 14.64 (3.37) | 15.40 (2.89) |
| **Internalising Difficulties** | | | | |
| range age 11 | 0 – 19 | 0 – 19 | 0 – 18 | 0 – 18 |
| mean age 11 (sd) | 3.01 (2.98) | 3.19 (3.20) | 4.31 (3.66) | 3.74 (3.41) |
| range age 14 | 0 – 19 | 0 – 19 | 0 – 19 | 0 – 18 |
| mean age 14 (sd) | 3.59 (3.29) | 3.66 (3.48) | 4.59 (3.80) | 4.18 (3.60) |
| **Externalising Difficulties** |  |  |  |  |
| range age 11 | 0 – 20 | 0 – 19 | 0 – 19 | 0 – 19 |
| mean age 11 (sd) | 4.23 (3.44) | 4.77 (3.71) | 5.61 (3.84) | 4.95 (3.76) |
| range age 14 | 0 – 19 | 0 – 19 | 0 – 18 | 0 – 20 |
| mean age 14 (sd) | 4.15 (3.43) | 4.49 (3.74) | 5.06 (3.86) | 4.76 (3.65) |
| **Bullying Behaviour** | | | |  |
| range age 11 | 0 – 5 | 0 – 5 | 0 – 5 | 0 – 5 |
| mean age 11 (sd) | 0.43 (0.91) | 0.56 (1.11) | 0.70 (1.13) | 0.53 (1.06) |
| range age 14 | 0 – 5 | 0 – 5 | 0 – 4.5 | 0 – 5 |
| mean age 14 (sd) | 0.30 (0.59) | 0.42 (0.75) | 0.47 (0.76) | 0.33 (0.66) |
| **Being victimised** | | | | |
| range age 11 | 0 – 5 | 0 – 5 | 0 – 5 | 0 – 5 |
| mean age 11 (sd) | 1.18 (1.50) | 1.38 (1.69) | 2.05 (1.84) | 1.53 (1.73) |
| range age 14 | 0 – 5 | 0 – 5 | 0 – 5 | 0 – 5 |
| mean age 14 (sd) | 0.69 (0.95) | 0.74 (0.97) | 1.06 (1.14) | 0.77 (1.04) |
| **Emotional Difficulties** | | | | |
| range age 11 | 0 – 10 | 0 – 10 | 0 – 10 | 0 – 10 |
| mean age 11 (sd) | 1.77 (1.92) | 1.80 (2.01) | 2.44 (2.23) | 2.08 (2.10) |
| range age 14 | 0 – 10 | 0 – 10 | 0 – 10 | 0 – 10 |
| mean age 14 (sd) | 1.97 (2.08) | 1.95 (2.16) | 2.39 (2.24) | 2.22 (2.25) |
| **Peer Problems** | | | | |
| range age 11 | 0 – 10 | 0 – 10 | 0 – 9 | 0 – 9 |
| mean age 11 (sd) | 1.24 (1.57) | 1.37 (1.70) | 1.87 (1.97) | 1.67 (1.89) |
| range age 14 | 0 – 10 | 0 – 9 | 0 – 10 | 0 – 10 |
| mean age 14 (sd) | 1.62 (1.74) | 1.71 (1.81) | 2.21 (2.13) | 1.96 (1.92) |
| **Drinking Behaviour** | | | | |
| range age 11 | 1 – 6 | 1 – 7 | 1 – 7 | 1 – 7 |
| mean age 11 (sd) | 1.03 (0.21) | 1.07 (0.38) | 1.08 (0.47) | 1.04 (0.25) |
| range age 14 | 1 – 7 | 1 – 5 | 1 – 7 | 1 – 7 |
| mean age 14 (sd) | 1.30 (0.67) | 1.37 (0.74) | 1.42 (0.83) | 1.24 (0.61) |
| **Binge Drinking Behaviour** | | | | |
| range age 11 | 1 – 6 | 1 – 4 | 1 – 6 | 1 – 4 |
| mean age 11 (sd) | 1.01 (0.15) | 1.02 (0.20) | 1.03 (0.30) | 1.02 (0.17) |
| range age 14 | 1 – 5 | 1 – 5 | 1 – 5 | 1 – 5 |
| mean age 14 (sd) | 1.13 (0.51) | 1.20 (0.58) | 1.23 (0.66) | 1.09 (0.40) |
| **Emotional Difficulties** | | | | |
| range age 11 | 0 – 10 | 0 – 10 | 0 – 10 | 0 – 10 |
| mean age 11 (sd) | 1.77 (1.92) | 1.80 (2.01) | 2.44 (2.23) | 2.08 (2.10) |
| range age 14 | 0 – 10 | 0 – 10 | 0 – 10 | 0 – 10 |
| mean age 14 (sd) | 1.97 (2.08) | 1.95 (2.16) | 2.39 (2.24) | 2.22 (2.25) |
| **Peer Problems** | | | | |
| range age 11 | 0 – 10 | 0 – 10 | 0 – 9 | 0 – 9 |
| mean age 11 (sd) | 1.24 (1.57) | 1.37 (1.70) | 1.87 (1.97) | 1.67 (1.89) |
| range age 14 | 0 – 10 | 0 – 9 | 0 – 10 | 0 – 10 |
| mean age 14 (sd) | 1.62 (1.74) | 1.71 (1.81) | 2.21 (2.13) | 1.96 (1.92) |

**Table S5**

*Pairwise comparisons resulting from each one-way ANOVA*

| ***Group 1*** | ***Group 2*** | ***N of group 1*** | ***N of group 2*** | | ***t*** | ***df*** | | ***p value*** | | ***Adjusted p value^a^*** | |  |
| --- | --- | --- | --- | --- | --- | --- | --- | --- | --- | --- | --- | --- |
| **Well-being at age 11** | | | | | | | | | | | |  |
| Equal | IDK | 9302 | 2122 | 6.98 | | 2913.378 | | <.001 | | <.001 | | |
| Equal | Poorer | 9302 | 527 | 12.14 | | 560.94 | | <.001 | | <.001 | | |
| Equal | Richer | 9302 | 1044 | 3.65 | | 1195.72 | | <.001 | | 0.002 | | |
| IDK | Poorer | 2122 | 527 | 7.78 | | 774.65 | | <.001 | | <.001 | | |
| IDK | Richer | 2122 | 1044 | -1.02 | | 1899.64 | | .308 | | >.999 | | |
| Poorer | Richer | 527 | 1044 | -7.67 | | 1070.34 | | <.001 | | <.001 | | |
| **Self-Esteem at age 11** | | | | | | | | | | | |  |
| Equal | IDK | 9302 | 2122 | 7.42 | | 2616.58 | | <.001 | | <.001 | | |
| Equal | Poorer | 9302 | 527 | 9.42 | | 526.28 | | <.001 | | <.001 | | |
| Equal | Richer | 9302 | 1044 | -1.42 | | 1134.44 | | .156 | | .936 | | |
| IDK | Poorer | 2122 | 527 | 5.54 | | 692.95 | | <.001 | | <.001 | | |
| IDK | Richer | 2122 | 1044 | -5.86 | | 1913.75 | | <.001 | | <.001 | | |
| Poorer | Richer | 527 | 1044 | -8.92 | | 876.20 | | <.001 | | <.001 | | |
| **Internalising difficulties at age 11** | | | | | | | | | | | |  |
| Equal | IDK | 9302 | 2122 | -8.93 | | 2741.85 | | <.001 | | <.001 | | |
| Equal | Poorer | 9302 | 527 | -7.91 | | 549.46 | | <.001 | | <.001 | | |
| Equal | Richer | 9302 | 1044 | -1.75 | | 1181.66 | | .081 | | .487 | | |
| IDK | Poorer | 2122 | 527 | -3.18 | | 750.81 | | .002 | | .009 | | |
| IDK | Richer | 2122 | 1044 | 4.32 | | 2072.26 | | <.001 | | <.001 | | |
| Poorer | Richer | 527 | 1044 | 5.85 | | 919.83 | | <.001 | | <.001 | | |
| **Externalising difficulties at age 11** | | | | | | | | | | | |  |
| Equal | IDK | 9302 | 2122 | -7.82 | | 2815.18 | | <.001 | | <.001 | | |
| Equal | Poorer | 9302 | 527 | -7.91 | | 556.81 | | <.001 | | <.001 | | |
| Equal | Richer | 9302 | 1044 | -4.31 | | 1174.63 | | <.001 | | <.001 | | |
| IDK | Poorer | 2122 | 527 | -3.49 | | 774.46 | | .001 | | .003 | | |
| IDK | Richer | 2122 | 1044 | 1.24 | | 1967.53 | | .214 | | >.999 | | |
| Poorer | Richer | 527 | 1044 | 4.06 | | 1000.34 | | <.001 | | <.001 | | |
| **Bullying at age 11** | | | | | | | | | | | |  |
| Equal | IDK | 9302 | 2122 | -3.88 | | 2817.37 | | <.001 | | .001 | | |
| Equal | Poorer | 9302 | 527 | -5.24 | | 556.69 | | <.001 | | <.001 | | |
| Equal | Richer | 9302 | 1044 | -3.73 | | 1194.05 | | <.001 | | .001 | | |
| IDK | Poorer | 2122 | 527 | -3.05 | | 761.72 | | .002 | | .014 | | |
| IDK | Richer | 2122 | 1044 | -0.86 | | 1981.99 | | .389 | | >.999 | | |
| Poorer | Richer | 527 | 1044 | 2.17 | | 1019.84 | | .030 | | .179 | | |
| **Victimisation at age 11** | | | | | | | | | | | |  |
| Equal | IDK | 9302 | 2122 | -8.47 | | 2836.84 | | <.001 | | <.001 | | |
| Equal | Poorer | 9302 | 527 | -10.51 | | 556.47 | | <.001 | | <.001 | | |
| Equal | Richer | 9302 | 1044 | -3.65 | | 1222.40 | | <.001 | | .002 | | |
| IDK | Poorer | 2122 | 527 | -5.80 | | 760.31 | | <.001 | | <.001 | | |
| IDK | Richer | 2122 | 1044 | 2.27 | | 2104.26 | | .023 | | .139 | | |
| Poorer | Richer | 527 | 1044 | 6.90 | | 960.09 | | <.001 | | <.001 | | |
| **Victimisation change scores between age 11 and 14** | | | | | | | | | | | |  |
| Equal | IDK | 9302 | 2122 | | 6.79 | | 2308.55 | | <.001 | | <.001 | |
| Equal | Poorer | 9302 | 527 | | 5.80 | | 458.77 | | <.001 | | <.001 | |
| Equal | Richer | 9302 | 1044 | | 2.09 | | 954.30 | | .037 | | .221 | |
| IDK | Poorer | 2122 | 527 | | 2.26 | | 621.82 | | .024 | | .143 | |
| IDK | Richer | 2122 | 1044 | | -2.45 | | 1614.30 | | .014 | | .086 | |
| Poorer | Richer | 527 | 1044 | | -3.73 | | 799.09 | | <.001 | | .001 | |

**Table S6**

*Pairwise comparisons resulting from the emotional difficulties, peer problems, drinking and binge drinking one-way ANOVAs*

| ***Outcome measure*** | ***Group 1*** | ***Group 2*** | ***N of group 1*** | ***N of group 2*** | ***t*** | ***df*** | ***p value*** | ***Adjusted p value^a^*** |
| --- | --- | --- | --- | --- | --- | --- | --- | --- |
| **Emotional difficulties at age 11** | | | | | | | | |
|  | Equal | IDK | 9302 | 2122 | -5.97 | 2819.27 | <.001 | <.001 |
|  | Equal | Poorer | 9302 | 527 | -6.65 | 555.32 | <.001 | <.001 |
|  | Equal | Richer | 9302 | 1044 | -0.42 | 1191.37 | .676 | >.999 |
|  | IDK | Poorer | 2122 | 527 | -3.34 | 757.34 | <.001 | .005 |
|  | IDK | Richer | 2122 | 1044 | 3.48 | 2030.02 | <.001 | .003 |
|  | Poorer | Richer | 527 | 1044 | 5.45 | 947.33 | <.001 | <.001 |
| **Peer problems at age 11** | | | | | | | | |
|  | Equal | IDK | 9302 | 2122 | -9.51 | 2673.78 | <.001 | <.001 |
|  | Equal | Poorer | 9302 | 527 | -7.15 | 548.85 | <.001 | <.001 |
|  | Equal | Richer | 9302 | 1044 | -2.78 | 1179.95 | .006 | .034 |
|  | IDK | Poorer | 2122 | 527 | -2.11 | 766.34 | .035 | .211 |
|  | IDK | Richer | 2122 | 1044 | 3.99 | 2153.03 | <.001 | <.001 |
|  | Poorer | Richer | 527 | 1044 | 4.65 | 910.64 | <.001 | <.001 |
| **Drinking age 11** | | | | | | | | |
|  | Equal | IDK | 9302 | 2122 | -0.65 | 2749.83 | .517 | >.999 |
|  | Equal | Poorer | 9302 | 527 | -2.36 | 516.82 | .018 | .111 |
|  | Equal | Richer | 9302 | 1044 | -2.64 | 1091.87 | .008 | .051 |
|  | IDK | Poorer | 2122 | 527 | -2.11 | 580.07 | .035 | .210 |
|  | IDK | Richer | 2122 | 1044 | -2.14 | 1479.28 | .033 | .197 |
|  | Poorer | Richer | 527 | 1044 | 0.72 | 848.13 | .471 | >.999 |
| **Binge drinking change between age 11 and 14** | | | | | | | | |
|  | Richer | Equal | 1044 | 9303 | 3.52 | 2891.16 | <.001 | .003 |
|  | Richer | Poorer | 1044 | 527 | -2.66 | 449.10 | .008 | .048 |
|  | Richer | IDK | 1044 | 2122 | -1.97 | 922.55 | .049 | .295 |
|  | Equal | Poorer | 9303 | 527 | -3.85 | 512.34 | <.001 | <.001 |
|  | Equal | IDK | 9303 | 2122 | -3.60 | 1170.34 | <.001 | .002 |
|  | Poorer | IDK | 527 | 2122 | 1.07 | 816.77 | .287 | >.999 |

**Supplemental Figures**

**Figure S2**

*PIIAF group (richer, equal, poorer, I don’t know; IDK) differences in bullying at age 11 as determined by an ANOVA.*

*Note.* This boxplot represents the median score of each PIIAF group and its interquartile range, lines represent the spread of the data and dots represent outliers for each PIIAF group. Poorer PIIAF group shows lower bullying than equal PIIAF and IDK PIIAF groups, but not than richer PIIAF. Equal PIIAF groups shows lower bullying than all PIIAF groups.
